# Growth capacity of a Wharton’s Jelly derived mesenchymal stromal cells tissue engineered vascular graft used for main pulmonary artery reconstruction in piglets

**DOI:** 10.3389/fbioe.2024.1360221

**Published:** 2024-02-23

**Authors:** Dominga Iacobazzi, Mohamed T. Ghorbel, Filippo Rapetto, Srinivas A. Narayan, Julia Deutsch, Tasneem Salih, Amy G. Harris, Katie L. Skeffington, Richard Parry, Giulia Parolari, Guillaume Chanoit, Massimo Caputo

**Affiliations:** ^1^ Translational Health Sciences, University of Bristol, Bristol, United Kingdom; ^2^ Department of Cardiac Surgery, Bristol Royal Hospital for Children, Bristol, United Kingdom; ^3^ Department of Paediatric Cardiology, Bristol Royal Hospital for Children, Bristol, United Kingdom; ^4^ Langford Clinical Veterinary Services, University of Bristol, Bristol, United Kingdom; ^5^ VetAgroSup—Veterinary Campus Lyon, Lyon, France

**Keywords:** right ventricular outflow tract reconstruction, tissue engineering, small intestinal submucosa, growing swine model, preclinical efficacy

## Abstract

**Background:** Surgical treatment of congenital heart defects affecting the right ventricular outflow tract (RVOT) often requires complex reconstruction and multiple reoperations due to structural degeneration and lack of growth of currently available materials. Hence, alternative approaches for RVOT reconstruction, which meet the requirements of biocompatibility and long-term durability of an ideal scaffold, are needed. Through this full scale pre-clinical study, we demonstrated the growth capacity of a Wharton’s Jelly derived mesenchymal stromal cells (WJ-MSC) tissue engineered vascular graft used in reconstructing the main pulmonary artery in piglets, providing proof of biocompatibility and efficacy.

**Methods:** Sixteen four-week-old Landrace pigs were randomized to undergo supravalvar Main Pulmonary Artery (MPA) replacement with either unseeded or WJ-MSCs-seeded Small Intestinal Submucosa-derived grafts. Animals were followed up for 6 months by clinical examinations and cardiac imaging. At termination, sections of MPAs were assessed by macroscopic inspection, histology and fluorescent immunohistochemistry.

**Results:** Data collected at 6 months follow up showed no sign of graft thrombosis or calcification. The explanted main pulmonary arteries demonstrated a significantly higher degree of cellular organization and elastin content in the WJ-MSCs seeded grafts compared to the acellular counterparts. Transthoracic echocardiography and cardiovascular magnetic resonance confirmed the superior growth and remodelling of the WJ-MSCs seeded conduit compared to the unseeded.

**Conclusion:** Our findings indicate that the addition of WJ-MSCs to the acellular scaffold can upgrade the material, converting it into a biologically active tissue, with the potential to grow, repair and remodel the RVOT.

## Introduction

Advances in paediatric cardiac surgery and postoperative care during the past few decades have led to more children with Congenital Heart Disease (CHDs) living longer with a better quality of life. However, the major hurdle of limited durability and decreased performance over time of currently used prosthetic solutions to correct defected hearts is still far from being addressed, thus considerably affecting young patients’ quality of life and the national healthcare systems finances (Tweddell et al., 2000; Brown et al., 2005; Selamet Tierney et al., 2005; Karamlou et al., 2006; Poynter et al., 2013; Vitanova et al., 2014). The impact of this problem has now widely been acknowledged by national and international health bodies and working groups. The National Institute for Health Research (NIHR), supported by the James Lind Alliance (JLA) Priority Setting Partnership (PSP) in congenital heart surgery, has identified the need to reduce the number of re-operations in children with CHDs as one of the major needs in cardiac surgery, and acknowledged progress in Tissue Engineering (TE) research as one of the top ten research priorities of CHDs (Drury et al., 2022). In this scenario, pre-clinical studies proving safety and efficacy of TE products are paramount for clinical translation, which can be very challenging, due to the disparity between the animal model disease and human complexity, or for the weakness of preclinical evidence often related to low sample size (Drude et al., 2021).

**FIGURE 1:**
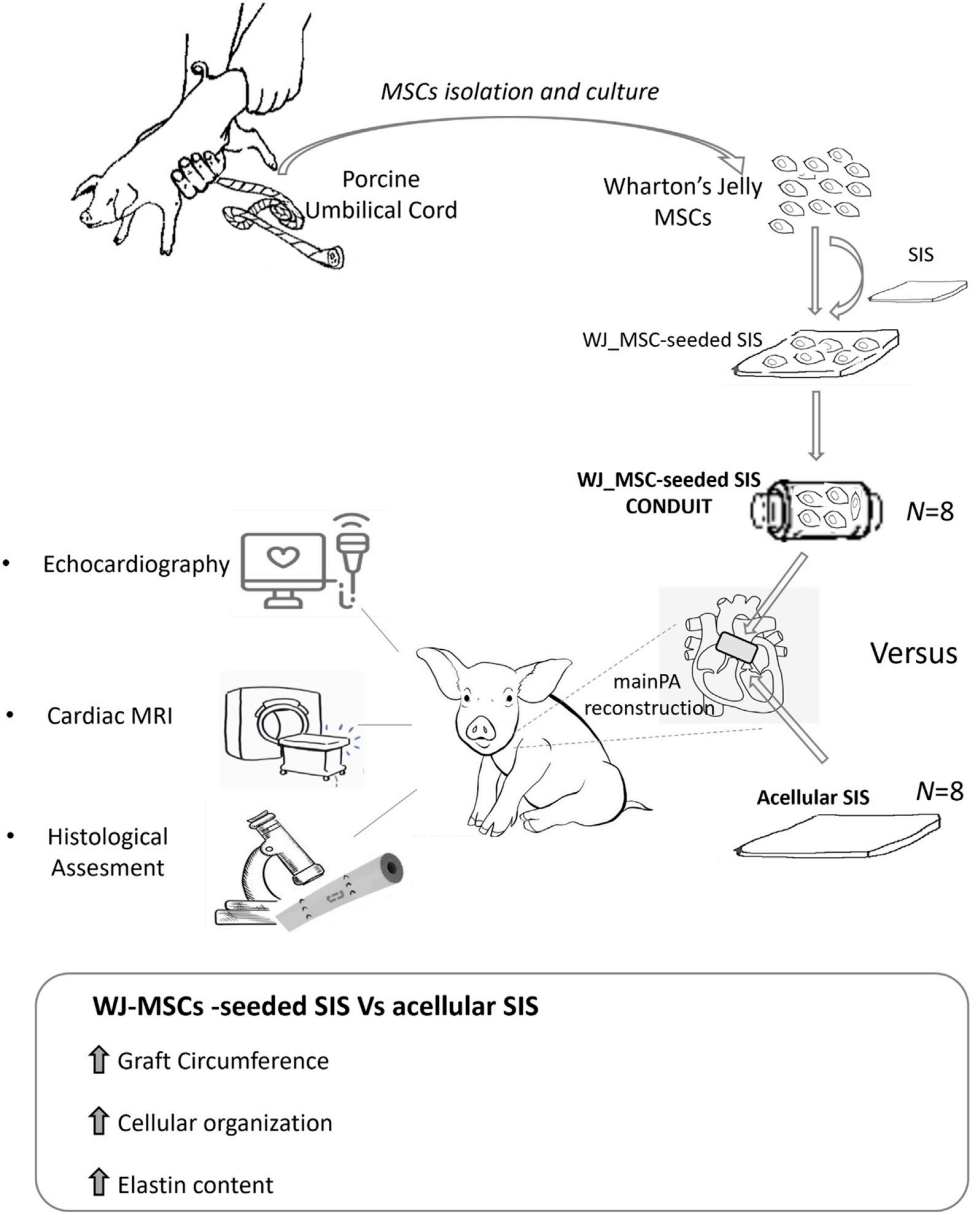
Visual abstract summarizing the experimental and results sections.

We have recently developed a TE vascular graft consisting of a Wharton’s Jelly derived mesenchymal stromal cells (WJ-MSC) seeded onto a Small Intestinal Submucosa (SIS), and tested it in a porcine model of main pulmonary artery (MPA) reconstruction as a first safety study. This preliminary investigation showed the safety, tissue remodelling and integration capacity of our TE product (named WJ-MSC SIS) within the host, as compared to the acellular scaffold (Rapetto et al., 2022). In this study we aim at testing the growing capacity and efficacy of our WJ-MSC-seeded SIS product by performing a full-scale pre-clinical study of MPA reconstruction in growing piglet (Figure 1).

## Methods

### Animals

Porcine umbilical cord samples were collected within 24 h after birth from Landrace female piglets (average weight 1.5 kg) sacrificed using Schedule 1, following the guidelines of the United Kingdom Home Office. Four-week-old female Landrace pigs were employed for the *in vivo* graft implantation studies under the United Kingdom Home Office ethical approval PP0950206. Animals were treated in accordance with the “Guide for the Care and Use of Laboratory Animals” published by the National Institutes of Health in 1996 and conforming to the “Animals (Scientific Procedures) Act” published in 1986.

### Porcine WJ-MSCs culture and graft cellularization

WJ-MSCs were isolated from umbilical cord of newborn female piglets by mechanical dissociation, as previously described (Iacobazzi et al., 2021). Briefly, the umbilical cord was cut open and 2–3 cm^2^ segments were removed from the inner gelatinous tissue. The finely minced tissues were distributed in a sterile petri dish and the culture medium poured slowly and gently, so as not to detach the fragments, which were then cultured in DMEM (Life Technologies) supplemented with 10% Hyclone Fetal Bovine Serum (FBS, Thermo Scientific) at 37°C in a humidified 5% CO2 incubator.

The isolated cells were fed with fresh medium every 3 days and expanded until passages 2 to 5. Fluorescence-activated cell sorting analysis was used to determine cell surface marker expression (Albertario et al., 2019; Iacobazzi et al., 2021). The following primary antibodies were used: 1:10 CD31-PE (Bio-Rad, Hercules, California), 1:600 CD44-APC (Thermo Fisher Scientific), 1:25 CD45-FITC (Bio-Rad), 1:10 CD73-APC (R and D Systems, Minneapolis, Minnesota), 1:20 CD90-PE (BioLegend, San Diego, California), and 1:5 CD105-PE (LSBio, Seattle, Washington). Analysis was performed on a NovoCyte flow cytometer (ACEA Bioscience, San Diego, California) using NovoExpress (ACEA Bioscience) for data collection and FlowJo (TreeStar, Ashland, Ohio) for analysis.

Expanded WJ-MSCs (passage 2 to passage 5) were seeded onto decellularized porcine SIS at a density of 2.5 × 10^5^/cm^2^ and cultured until graft maturation, according to the protocol previously optimized by our group (Iacobazzi et al., 2018; Albertario et al., 2019; Iacobazzi et al., 2021), which involved a culture under static conditions for 3 days, followed by the transferring into an InBreath bioreactor (Harvard Apparatus, United States), where it was stitched to its rotating arm as to fashion a conduit-shape, with the cells facing the outer side of the graft, and cultured for additional 3 days.

### *In vivo* experiments

A total of 16 four-week-old healthy Landrace female piglets (mean weight 20.5 ± 4.1 kg) were employed in this study.

**FIGURE 2:**
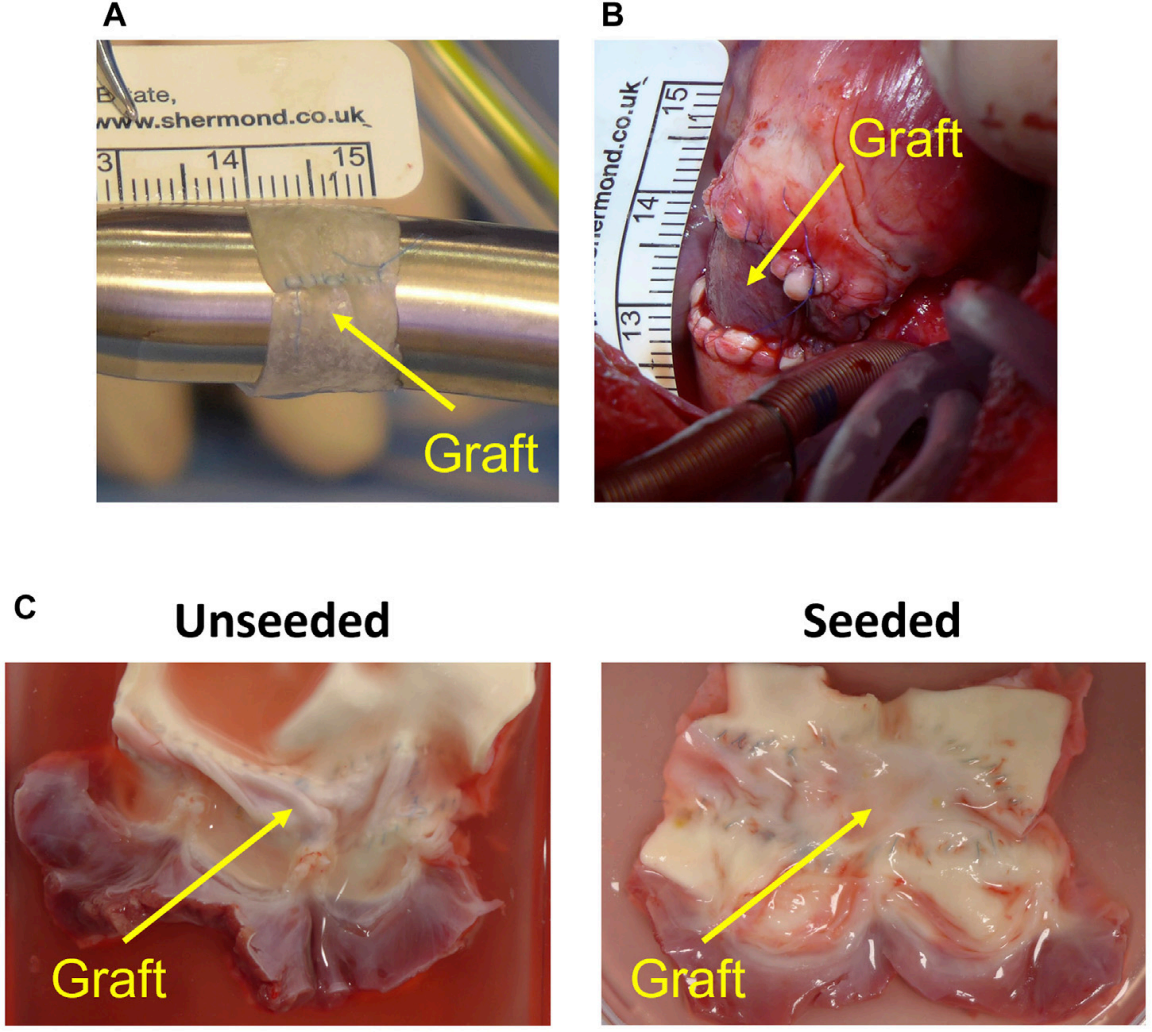
Graft preparation and *in vivo* implantation. **(A)** Circumferential graft created from patch of small intestinal submucosa (running longitudinal suture visible). **(B)** Graft after implantation on main pulmonary artery. **(C)** Representative image of the explanted acellular and cell-engineered conduit.

On the day of surgery, all animals were premedicated with Ketamine 10 micrograms/kg and/or Dexmedetomidine 15 micrograms/kg injected intramuscularly; general anesthesia was induced with intravenous Propofol and maintained with Isoflurane in oxygen. A continuous intravenous infusion of Pancuronium Bromide (0.1 mg/kg/h) was used to achieve neuromuscular blockade. Immediately before starting the surgical procedure, transthoracic echocardiography (TTE) was performed. Following this, a circumferential graft was created from a rectangular SIS patch (length = 10–12 mm, circumference = MPA diameter as measured by TTE, multiplied by 3) as shown in Figure 2A 8 animals were randomized to receive a graft made from commercially available SIS, whereas 8 animals received a WJ-MSC seeded SIS graft; the surgeon was blinded to the graft seeding.

The heart was accessed by median sternotomy, and cardiopulmonary bypass was established by cannulating the ascending aorta, the superior vena cava directly and the inferior vena cava via the right atrial appendage. On the beating heart, an 8 mm segment of the MPA was excised, leaving the native pulmonary valve and annulus intact. The previously prepared circumferential conduit was then implanted as an interposition graft on the MPA, distal to the pulmonary valve (Figure 2B). Animals were extubated on the day of surgery and received intensive care for 24 h postoperatively. Pain was managed with opioids and NSAIDs; non-invasive blood pressure, heart rate, oxygen saturation, temperature and chest drain output were checked every 2–4 h during this time. All animals underwent TTE and cardiovascular magnetic resonance immediately before surgery and 6 months postoperatively. Peak velocities across the MPA and the RVOT were measured by TTE; MPA flow was measured by CMR. Animals were euthanized with an intravenous injection of 150 mg/kg of Pentobarbital sodium after completing the 6-month follow-up.

### Histology

Explanted samples (Figure 2C) were washed in PBS and fixed overnight with 4% paraformaldehyde at 4°C. Fixed tissues were processed in a Thermo Excelsior AS (Thermo Fisher Scientific, Waltham, Massachusetts, United States) and embedded with a Thermo HistoStar (Thermo Fisher Scientific) machine. Five-micrometer-thick sections were cut using a Shandon Finesse 325 microtome (Thermo Fisher Scientific). Slides were stored at 37°C overnight to dry completely before staining. Hematoxylin and eosin, and Van Gieson’s stainings were performed using a Shandon Varistain 24–4 (Thermo Fisher Scientific) automated machine. Von Kossa staining was carried out using a Silver plating kit (*In Vitro* Diagnostic Medical Device, Darmstadt, Germany) for the detection of microcalcification. Collagen and elastin contents of the explants in the Van Gieson’s staining were quantified using ImageJ software via color deconvolution, to separate and convert the collagen and elastic fibers to the mean Gray value of the pink and purple and colour intensity, respectively. Threshold values were set for each channel and area-based analysis was used to extract and quantify the regions of interest (ROIs) from the image. Results were expressed as proportion of area occupied by collagen or elastin within the graft tissue.

### Immunohistochemistry

Paraffin-embedded sections were deparaffinized by two changes of clerene and rehydrated through an alcohol gradient. A heated antigen retrieval with 10 mM citrate buffer pH 6.0 was performed. Samples were blocked with 10% goat serum (Sigma-Aldrich, St. Louis, Missouri, United States) in phosphate-buffered saline for 30 min at room temperature and incubated with the unconjugated primary antibodies (α-SMA, 1:100, Sigma-Aldrich; Isolectin B4-Biotin 1:100, Life Technologies, Carlsbad, California, United States; Elastin, 1:100, Santa Cruz Biotechnology) overnight at 4°C. Fluorophore-conjugated (Alexa Fluor 488 and Alexa Fluor 546, 1:400, Life Technologies) or chromogen-conjugated (HRP-, 1:1000, R&D System) secondary antibodies were incubated on the sections for 1 h at room temperature in the dark. Nuclei were counterstained with 4′,6-diamidino-2-phenylindole (1:1000; Sigma-Aldrich). Slides were mounted with Hardset mounting medium (Vectashield). Images were taken with a Zeiss Observer.Z1 fluorescent microscope. ImageJ software was used to quantify the smooth muscle actin (SMA) and elastin expression in the tissue sections.

### Mechanical testing

Fresh explanted grafts (5 mm × 15 mm) were analysed for mechanical properties using an Instron 3343B machine (Instron, United Kingdom) with a 100 N load cell and pneumatic grips. Crosshead speed was 10 mm/min. Samples were measured for tensile stress at break using Bluehill software (Instron, United Kingdom).

### Statistical analysis

Data was tested for normality and analysed using one-way ANOVA followed by a post-hoc test. *p* ≤ 0.05 was considered statistically significant.

Continuous variables are expressed as mean ± standard deviation (SD). Data were recorded and subsequently tabulated with Microsoft Excel (VR Microsoft Corp, Redmond, Washington, United States). The statistical analysis were conducted using RStudio version 1.2.5042 (RStudio: Integrated Development for R. RStudio, Inc., Boston, Massachusetts, United States).

## Results

### *In vivo* assessment

**FIGURE 3:**
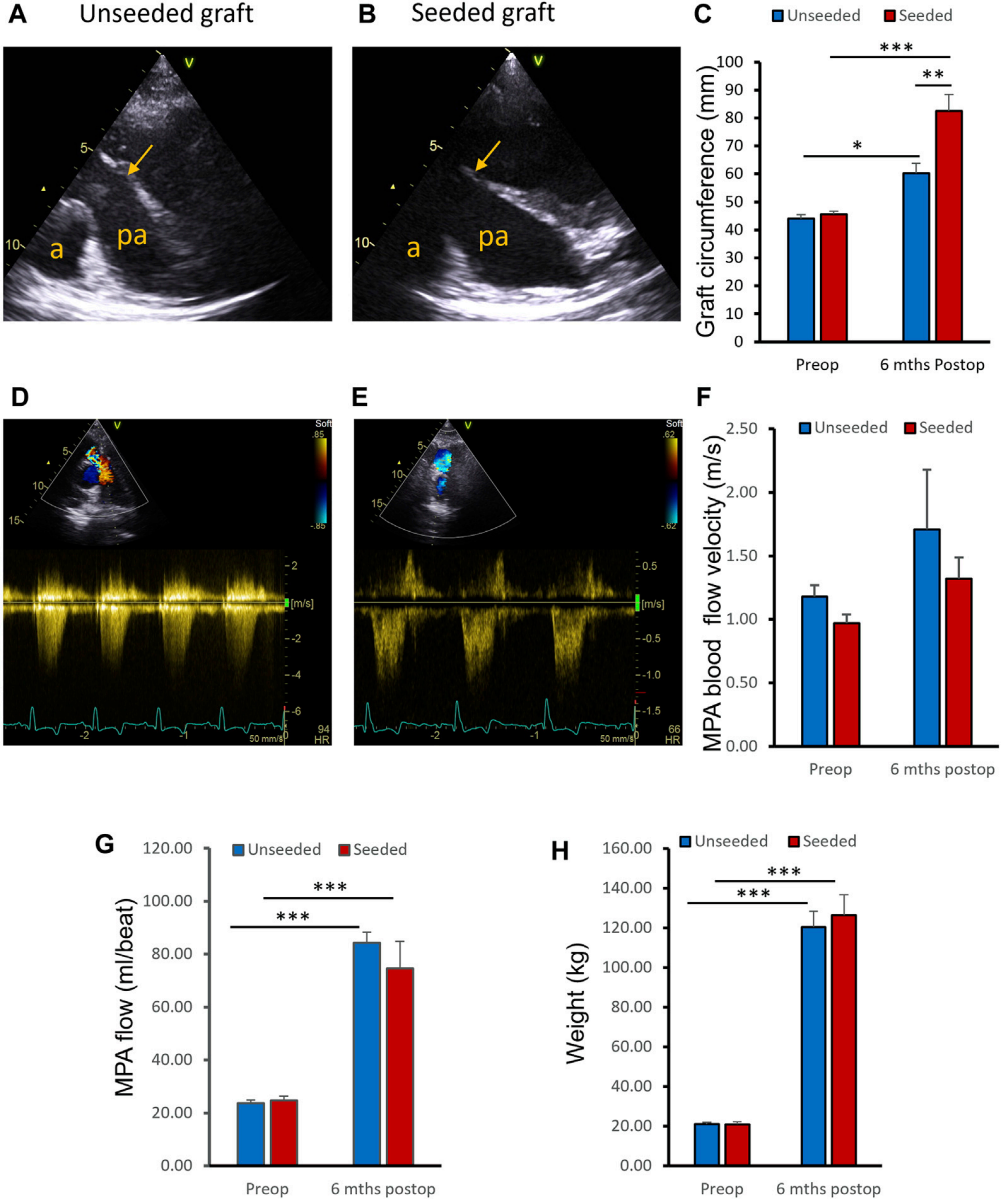
*In vivo* assessments. **(A, B)** Representative ultrasound images of the pulmonary arteries (PA) of unseeded and WJ-MSC seeded SIS grafts at 6 months post-surgery. Arrows indicate level of graft insertion (a, aorta; pa, pulmonary artery). **(C)** Circumference of the WJ-MSC seeded SIS and unseeded grafts at implantation and 6 months post-surgery. **(D, E)** Representative images of Colour Doppler blood velocities in PA of unseeded and cell-seeded groups. **(F)** Blood flow peak velocities through the PA of unseeded and WJ-MSC seeded groups pre-surgery and at 6 months of follow-up. **(G)** MPA flow measurements by cardiovascular magnetic resonance at pre-operation baseline and at 6-month follow-up, in unseeded and seeded animals. **(H)** Pigs’ weight pre-surgery and at 6 months of follow-up. ANOVA with *post-hoc* testing were used [n = 8, mean ± (SD), * *p* < 0.05, ** *p* < 0.01, *** *p* < 0.001].

Operated pigs grew at a normal rate and there was no significant difference in body weight gain between animals implanted with WJ-MSC seeded SIS or unseeded grafts at 6 months post-surgery (Figure 3H). Transthoracic Echocardiography showed grafts were patent in both groups (Figures 3A, B). At the 6 months assessment, the circumference of the WJ-MSC seeded SIS grafts showed 81% increase compared to pre-operation. In contrast, the circumference of the unseeded grafts showed only 36.8% increase compared to pre-operation (Figure 3C). Additionally, at 6 months post-surgery the circumference of the WJ-MSC SIS grafts was 36.9% longer than the unseeded ones (Figure 3C). Echocardiography demonstrated no significant difference in peak velocity across the MPA graft at 6 months postoperatively compared to baseline, both in the seeded and unseeded groups (Figures 3D–F). Blood flow in both groups showed a significant increase at follow-up compared to pre-operation baseline (Figure 3G). However, no significant difference in blood flow was observed between unseeded and seeded groups at the 6 month follow-up time point (Figure 3G).

### Histological and mechanical *ex vivo* analysis

At macroscopic inspection, explanted grafts presented a smooth luminal surface with no signs of obstruction or tissue calcification and degradation in both groups (Figure 2C).

**FIGURE 4:**
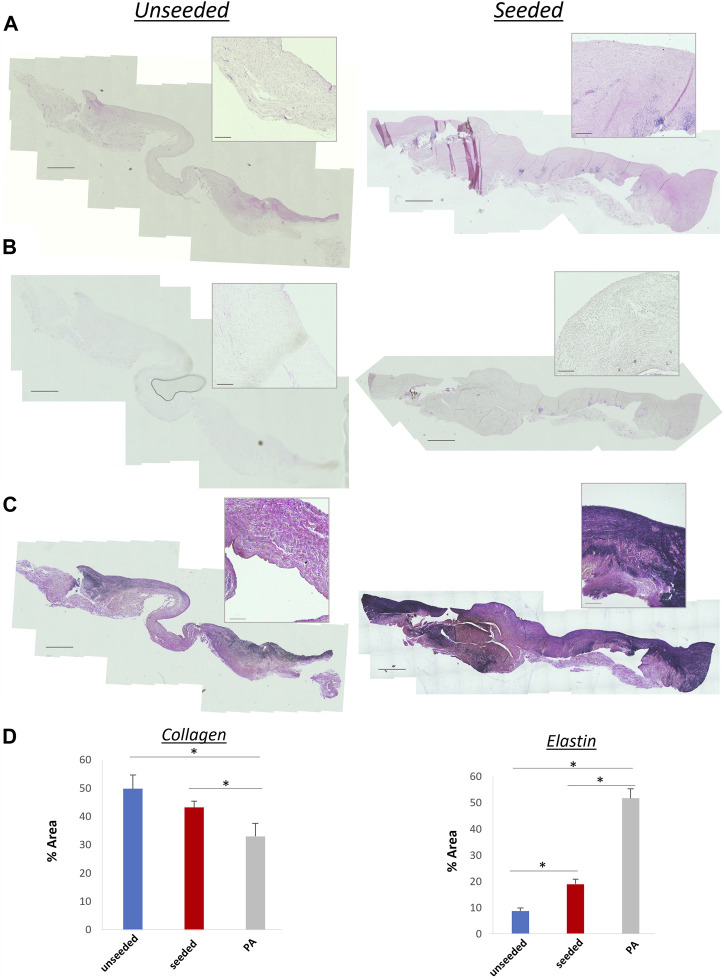
**(A)** Hematoxylin and eosin staining showing extensive nucleation throughout the structure of seeded and unseeded grafts. **(B)** Von Kossa staining showing no calcification in the grafts. **(C)** Van Gieson staining of collagen (pink) and elastin (purple) content of the grafts. A higher content of elastin, a major extracellular matrix component of the pulmonary artery, is visible in the seeded graft. **(D)** Bar plots showing collagen and elastin concentration in unseeded/seeded grafts and in the native MPA [n = 8, mean ± (SD), *p < 0.05, **p < 0.005]. Scale bars = 1,000 μm, 50 µm higher magnification.

**FIGURE 5:**
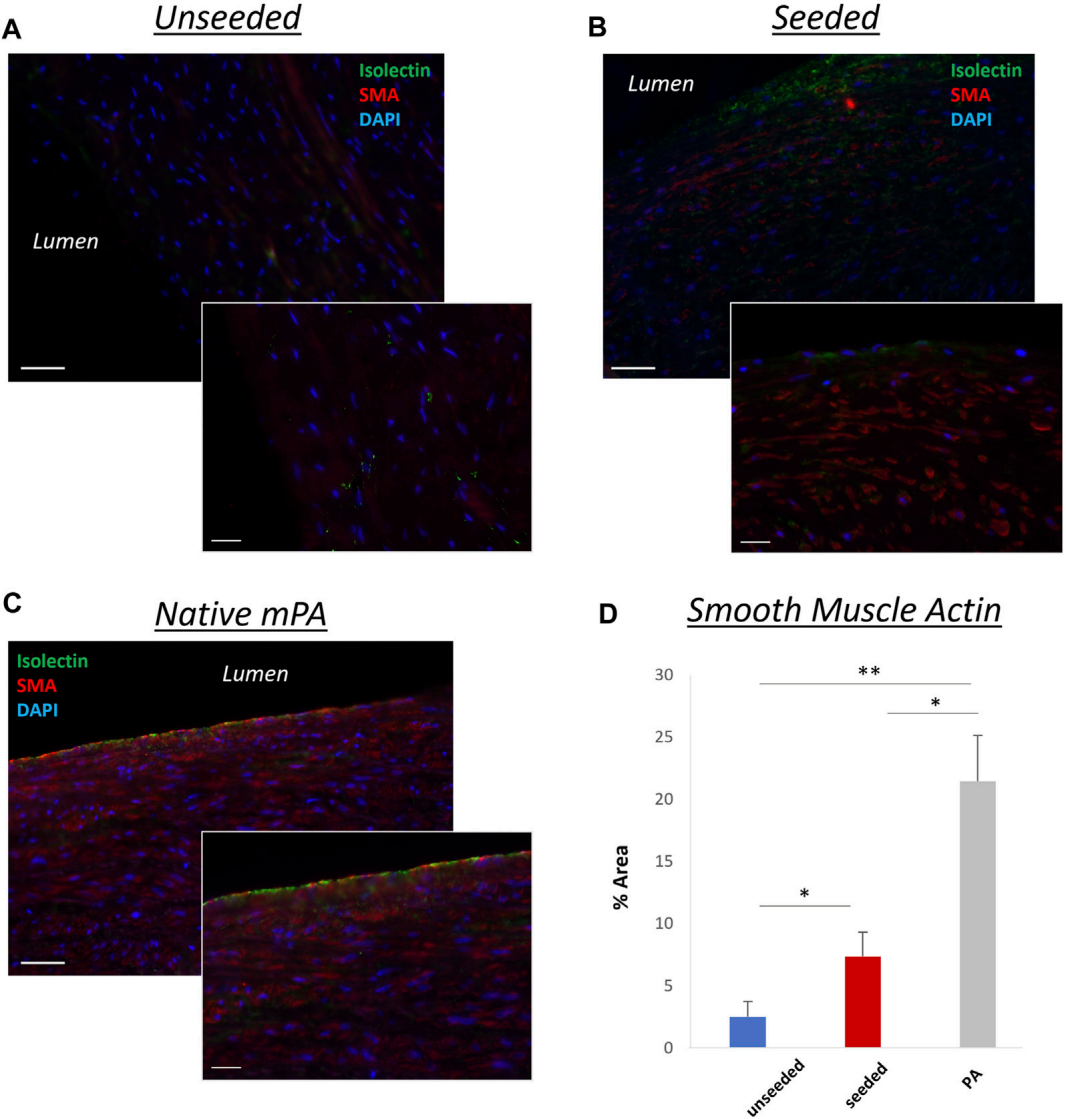
Representative images showing longitudinal sections of the grafts. A highly organized layer of smooth muscle cells [smooth muscle actin (SMA)–positive, red] and a newly formed layer of endothelial cells (isolectin-positive, green), similar to the native MPA **(C)**, were more pronounced in the WJ-MSC seeded SIS graft **(B)**, compared to the unseeded graft **(A)**. 40,6-diamidino-2-phenylindole was used to mark nuclei (blue). **(D)** Bar chart showing the different SMA concentrations in unseeded/seeded grafts and in native MPA [n = 8, mean ± (SD), *p < 0.05, **p < 0.005]. Scale bars = 1,000 μm, 50 µm higher magnification (smaller image).

**FIGURE 6:**
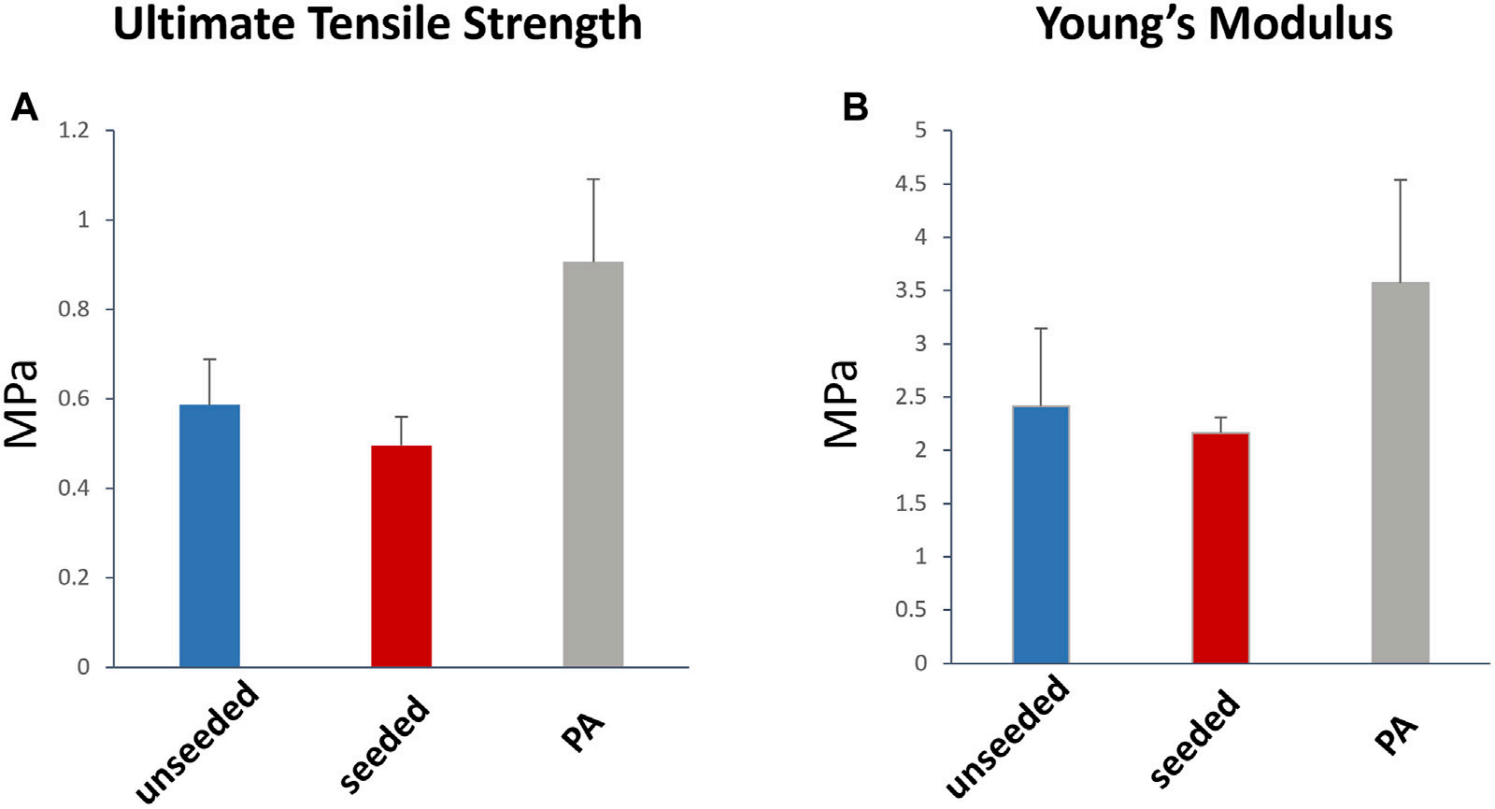
Tensile strength and elasticity of the explanted graft. Ultimate Tensile Strength **(A)** and Young’s modulus **(B)** were not significantly different between the WJ-MSC seeded and unseeded SIS scaffold, and the native MPA. MPa, megapascal.

The histological examination of the explanted grafts confirmed the previous finding of structural superiority of the seeded conduit, compared to the acellular counterpart. Hematoxylin and Eosin staining demonstrated a higher degree of nucleation throughout the WJ-MSC seeded SIS conduit (Figure 4A), with this displaying better cellular repopulation and organization, as confirmed by immunohistochemical staining which showed a newly formed layer of endothelial cells on the luminal side and a significantly higher number of SMA-stained cells repopulating the neo-tunica media (Figures 5A–D). Furthermore, Van Gieson staining showed a significantly higher content of elastin, a major extracellular matrix component of the pulmonary artery, in the seeded group compared to the unseeded (Figures 4C, D). Additionally, no signs of calcification were detected in either group (Figure 4B). Furthermore, no significant difference in the ultimate tensile strength and in Elastic Modulus (Young’s modulus) was noted when comparing the three groups, suggesting that both the seeded and unseeded grafts achieved mechanical properties similar to those of the native MPA (Figures 6A, B).

## Discussion

This study shows for the first time the growth capacity of a WJ-MSC-tissue engineered vascular graft used in reconstructing the main pulmonary artery in piglets. A porcine large animal model was used because of its cardiovascular system similarity to human’s. The rapid growth of the Landrace piglet made it ideal to assess growth in the 6 months follow-up period. This full scale pre-clinical study demonstrated the safety and efficacy of our novel tissue engineered conduit (made by WJ-MSCs-seeded SIS) for use *in vivo* RVOT reconstruction. It follows-up our previous safety investigation, in which we validated our porcine model of main Pulmonary Artery reconstruction and preliminarily tested in a small number of animals the safety and biocompatibility of our developed WJ-MSCs-seeded SIS (Rapetto et al., 2022).

The results of our *in vivo* study have major clinical implications for the treatment of paediatric heart defect conditions, such as ToF/Pulmonary Atresia, whereby RVOT reconstruction is needed. Indeed, they show that the WJ-MSC-tissue engineered vascular graft is a reliable biomaterial for main PA reconstruction that exhibits superior growth potential, compared to the acellular counterpart. These findings were supported by both imaging and histological analysis, and are in line with other works whereby progenitor cell-based grafts were shown to produce more organized elastin fibers when implanted in an *in vivo* model of jugular replacements in lambs (Swartz et al., 2005; Liu et al., 2007). Interestingly, in another study evaluating the remodeling capacity of cell free-silk vascular scaffold in rat aortae, such elastin increase was attributed to the MSCs-secreted Extracellular Vesicles (EV) rather than to the cells themselves (Cunnane et al., 2020a). We cannot exclude that a similar mechanism occurs with our WJ-MSCs seeded SIS, however this goes beyond the scope of this study, although worth it future investigation. Additionally, transthoracic echocardiography demonstrated that the circumference of the cell-seeded grafts significantly increased in comparison to the unseeded group. The histology showed a more organized cellular structure and higher elastin content in the seeded group compared to the unseeded.

We have previously shown the efficacy of a SIS scaffold recellularised with perinatal stem cells to reconstruct the left PA and its capacity to grow and remodel in piglet (Ghorbel et al., 2019). Here, we show the efficacy of a biomaterial produced by the same approach to reconstruct the main PA. Similar promising results were reported by Syedain *et al.* who demonstrated growth capacity and normal function of a TEVG implanted in a growing lamb model and followed up for 50 weeks (Syedain et al., 2016). One advantage of our approach over Syedain *et al.*’s is that we did not administer any anticoagulant to animals during the follow-up period. However, Syedain et al. had to administer heparin twice-daily for the 50 weeks duration of the experiment (Syedain et al., 2016). Positive outcome of vascular remodeling using stomal cell-derived vascular graft was also observed by Cunnane *et al.* in a sheep model of carotid interposition. Their developed seeded graft remained patent and exhibited signs of initiated neo-tissue formation throughout the length of the graft after 10 weeks (Cunnane et al., 2020b).

The regeneration of the tunica media is crucial in large diameter blood vessel tissue engineering. Our findings show a higher number of SMA-stained cells repopulating the neo-tunica media of the implanted WJ-MSC-seeded grafts compared to the unseeded. Furthermore, they show higher content of elastin in the WJ-MSC-seeded group compared to the unseeded. Our stem cell-seeding approach seems to support the formation of a healthy neo-tunica media, most likely by triggering the proliferation of recipient VSMCs after implantation and attracting them to the implanted graft. No signs of calcification were detected in either group.

These promising results demonstrate the safety, efficacy and reliability of WJ-MSC-tissue engineered vascular graft in reconstructing the main PA, which are fundamental requirements in the evaluation, by regulatory bodies, of an ATMP for clinical trial. Our findings are paving the way to the first-in-human clinical trial assessing the WJ-MSC-tissue engineered vascular graft to reconstruct the RVOT in infants with Tetralogy of Fallot/Pulmonary Atresia. The last decade has witnessed enormous progresses in the field of Tissue Engineering, with stem cells-seeded biocompatible scaffolds holding great promise for a new effective way to overcome the limitations of the currently used grafts in patients with CHD undergoing heart surgery (Khademhosseini and Langer, 2016; Jarrell et al., 2020). Our study is another example proving the suitability of this approach for corrective paediatric heart surgery.

### Study limitations

Despite the porcine species being a widely accepted model in cardiovascular research, owing to its similarity to humans in terms of cardiovascular anatomy and physiology, it has to be noted that the surgical procedures, although comparable to the ones performed in the defected human counterpart, are conducted on healthy animals without congenital malformations, making them a suboptimal test case for longer-term graft validation, due to the differences in pressure, flow and stress on the graft.

We also acknowledge the absence of a sham surgery control group, which, due to high costs and our compliance to the 3Rs principle of reducing the animal units as much as possible, was not included in this study.

## Data Availability

The raw data supporting the conclusion of this article will be made available by the authors, without undue reservation.

## References

[B1] Albertario A. Swim M. M. Ahmed E. M. Iacobazzi D. Yeong M. Madeddu P. (2019). Successful reconstruction of the right ventricular outflow tract by implantation of thymus stem cell engineered graft in growing swine. JACC Basic Transl. Sci. 4 (3), 364–384. 10.1016/j.jacbts.2019.02.001 31312760 PMC6609916

[B2] Brown J. W. Ruzmetov M. Rodefeld M. D. Vijay P. Turrentine M. W. (2005). Right ventricular outflow tract reconstruction with an allograft conduit in non-ross patients: risk factors for allograft dysfunction and failure. Ann. Thorac. Surg. 80 (2), 655–664. 10.1016/j.athoracsur.2005.02.053 16039222

[B3] Cunnane E. M. Lorentz K. L. Ramaswamy A. K. Gupta P. Mandal B. B. O'Brien F. J. (2020a). Extracellular Vesicles enhance the remodeling of cell-free silk vascular scaffolds in rat aortae. ACS Appl. Mater Interfaces 12 (24), 26955–26965. 10.1021/acsami.0c06609 32441910 PMC12039313

[B4] Cunnane E. M. Lorentz K. L. Soletti L. Ramaswamy A. K. Chung T. K. Haskett D. G. (2020b). Development of a semi-automated, bulk seeding Device for large animal model implantation of tissue engineered vascular grafts. Front. Bioeng. Biotechnol. 8, 597847. 10.3389/fbioe.2020.597847 33195168 PMC7644804

[B5] Drude N. I. Martinez Gamboa L. Danziger M. Dirnagl U. Toelch U. (2021). Improving preclinical studies through replications. Elife 10, e62101. 10.7554/elife.62101 33432925 PMC7817176

[B6] Drury N. E. Herd C. P. Biglino G. Brown K. L. Coats L. Cumper M. J. (2022). Research priorities in children and adults with congenital heart disease: a James Lind alliance priority setting partnership. Open Heart 9 (2), e002147. 10.1136/openhrt-2022-002147 36600635 PMC9843188

[B7] Ghorbel M. T. Jia H. Swim M. M. Iacobazzi D. Albertario A. Zebele C. (2019). Reconstruction of the pulmonary artery by a novel biodegradable conduit engineered with perinatal stem cell-derived vascular smooth muscle cells enables physiological vascular growth in a large animal model of congenital heart disease. Biomaterials 217, 119284. 10.1016/j.biomaterials.2019.119284 31255979 PMC6658806

[B8] Iacobazzi D. Rapetto F. Albertario A. Swim M. M. Narayan S. Skeffington K. (2021). Wharton's jelly-mesenchymal stem cell-engineered conduit for pediatric translation in heart defect. Tissue Eng. Part A 27 (3-4), 201–213. 10.1089/ten.tea.2020.0088 32571164

[B9] Iacobazzi D. Swim M. M. Albertario A. Caputo M. Ghorbel M. T. (2018). Thymus-derived mesenchymal stem cells for tissue engineering clinical-grade cardiovascular grafts. Tissue Eng. Part A 24 (9-10), 794–808. 10.1089/ten.tea.2017.0290 29054134

[B10] Jarrell D. K. Vanderslice E. J. VeDepo M. C. Jacot J. G. (2020). Engineering myocardium for heart regeneration-advancements, considerations, and future directions. Front. Cardiovasc Med. 7, 586261. 10.3389/fcvm.2020.586261 33195474 PMC7588355

[B11] Karamlou T. Blackstone E. H. Hawkins J. A. Jacobs M. L. Kanter K. R. Brown J. W. (2006). Can pulmonary conduit dysfunction and failure be reduced in infants and children less than age 2 years at initial implantation? J. Thorac. Cardiovasc Surg. 132 (4), 829–838.e5. 10.1016/j.jtcvs.2006.06.034 17000294

[B12] Khademhosseini A. Langer R. (2016). A decade of progress in tissue engineering. Nat. Protoc. 11 (10), 1775–1781. 10.1038/nprot.2016.123 27583639

[B13] Liu J. Y. Swartz D. D. Peng H. F. Gugino S. F. Russell J. A. Andreadis S. T. (2007). Functional tissue-engineered blood vessels from bone marrow progenitor cells. Cardiovasc Res. 75 (3), 618–628. 10.1016/j.cardiores.2007.04.018 17512920

[B14] Poynter J. A. Eghtesady P. McCrindle B. W. Walters H. L. Kirshbom P. M. Blackstone E. H. (2013). Association of pulmonary conduit type and size with durability in infants and young children. Ann. Thorac. Surg. 96 (5), 1695–1702. 10.1016/j.athoracsur.2013.05.074 23972424

[B15] Rapetto F. Iacobazzi D. Narayan S. A. Skeffington K. Salih T. Mostafa S. (2022). Wharton's jelly-mesenchymal stem cell-engineered conduit for pulmonary artery reconstruction in growing piglets. JACC Basic Transl. Sci. 7 (3), 207–219. 10.1016/j.jacbts.2021.11.013 35411313 PMC8993765

[B16] Selamet Tierney E. S. Gersony W. M. Altmann K. Solowiejczyk D. E. Bevilacqua L. M. Khan C. (2005). Pulmonary position cryopreserved homografts: durability in pediatric Ross and non-Ross patients. J. Thorac. Cardiovasc Surg. 130 (2), 282–286. 10.1016/j.jtcvs.2005.04.003 16077388

[B17] Swartz D. D. Russell J. A. Andreadis S. T. (2005). Engineering of fibrin-based functional and implantable small-diameter blood vessels. Am. J. Physiol. Heart Circ. Physiol. 288 (3), H1451–H1460. 10.1152/ajpheart.00479.2004 15486037

[B18] Syedain Z. Reimer J. Lahti M. Berry J. Johnson S. Tranquillo R. T. (2016). Tissue engineering of acellular vascular grafts capable of somatic growth in young lambs. Nat. Commun. 7, 12951. 10.1038/ncomms12951 27676438 PMC5052664

[B19] Tweddell J. S. Pelech A. N. Frommelt P. C. Mussatto K. A. Wyman J. D. Fedderly R. T. (2000). Factors affecting longevity of homograft valves used in right ventricular outflow tract reconstruction for congenital heart disease. Circulation 102 (19), III130–III135. 10.1161/01.cir.102.suppl_3.iii-130 11082375

[B20] Vitanova K. Cleuziou J. Horer J. Kasnar-Samprec J. Vogt M. Schreiber C. (2014). Which type of conduit to choose for right ventricular outflow tract reconstruction in patients below 1 year of age?dagger. Eur. J. Cardiothorac. Surg. 46 (6), 961–966. 10.1093/ejcts/ezu080 24616389

